# Pancreatic stump closure after pancreatoduodenectomy in elderly patients: a retrospective clinical study

**DOI:** 10.1007/s40520-016-0657-8

**Published:** 2016-11-11

**Authors:** Claudio Mauriello, Andrea Polistena, Claudio Gambardella, Ernesto Tartaglia, Michele Orditura, Ferdinando De Vita, Luigi Santini, Nicola Avenia, Giovanni Conzo

**Affiliations:** 1 Division of General and Oncologic Surgery, Department of Anesthesiologic, Surgical and Emergency Sciences, School of Medicine, Second University of Naples, Via Sergio Pansini 5, 80131 Naples, Italy; 2 Medical School, General Surgery and Surgical Specialties Unit, S. Maria University Hospital, University of Perugia, Terni, Italy; 3 Division of Medical Oncology, Department of Internal and Experimental Medicine “F. Magrassi”, School of Medicine, Second University of Naples, Via Sergio Pansini 5, 80131 Naples, Italy

**Keywords:** Pancreatoduodenectomy, Pancreatic duct occlusion, Pancreatic fistula, Elderly patients, Pancreatic cancer

## Abstract

**Background:**

Pancreatic fistula (PF) after pancreatoduodenectomy (PD) represents the major source of morbidity. Derivative procedures are preferred by pancreatic surgeons, but the optimal management of remnant pancreatic stump remains controversial.

**Aims:**

The purpose of this retrospective study is to evaluate the efficacy and safety of pancreatic stump closure in selected elderly patients (>65 years).

**Methods:**

Clinical data of 44 PD undergone mechanical closure of the pancreatic stump performed between 2001 and 2014 in two department of general and oncologic surgery were retrospectively collected. Considering the age, patients were divided into two groups: 21 patients of less than 65 years (Group A) and 23 patients of more than 65 years (Group B).

**Results:**

A soft pancreatic parenchyma with a not-dilated duct (diameter <3 mm) was reported in all the 44 patients. A grade-A PF, which did not required further treatments, developed in 20 cases (45.4%; 13 in group A and 7 in group B; *p* < 0.05), grade-B in 5 patients (11.4%; 3 in group A and 2 in group B; statistically not significant) and a grade-C PF was observed only in one patient (2.2%; 1 in group A and 0 in group B).

**Discussion:**

In selected “high risk” elderly patients (>65 years) with soft pancreatic texture, the closure of the pancreatic stump can be a useful tool in the surgical armamentarium with the aim to reduce the incidence of age-related complications.

**Conclusions:**

Prospective randomized controlled trial to better evaluate PF risk factors is needed.

## Introduction

The ductal adenocarcinoma of pancreas represents the fourth cause of cancer-related death in the world and, at the time of presentation, almost 50% of the patients have distant metastases [1].

When a resection is possible, surgery plus adjuvant chemotherapy is the treatment of choice in order to increase the chances for long-term survival, that unfortunately still remains of 5% after 5 years from diagnosis [2]. The most safe and widely performed procedure is pancreatoduodenectomy (PD), with a mortality <5% but with high morbidity rates up to 51% [3]. Although better elective surgery, enhanced resuscitation and intensive care, early diagnosis and treatment of complications, pancreatic fistula (PF) still remains the major source of morbidity (0–25%) and the optimal management of pancreatic stump is still theme of debate [4–6]. Conservative management of PF is often preferred thanks to the improvements in diagnostic and interventional radiological tools, but abdominal abscess, hemorrhage, peritonitis and sepsis are still common sequelae associated with a high mortality (about 40%), especially in elderly patients (>65 years) with life-threatening postoperative fistulas [7].

The major prognostic factors are texture of the pancreatic parenchyma and size of main pancreatic duct, together with surgeon experience, intraoperative blood loss, operation time, pancreatic anastomotic technique, jaundice and use of somatostatin. Comparing various anastomosis techniques with pancreatic stump closure many authors demonstrated the advantages of derivative procedures, even if controversial results have been reported [8–12].

The purpose of this retrospective study is to evaluate the efficacy and safety of pancreatic stump closure in selected “high risk” elderly patients (>65 years) as an alternative to the anastomotic technique.

## Patients and methods

The clinical data of 44 patients who underwent PD with closure of the pancreatic stump for neoplasms of pancreatic head or of the periampullary region in two surgical units of general and oncologic surgery between 2001 and 2014 were collected by two of the authors (C.M., C.G.) and retrospectively reviewed. These two units are medium volume for pancreatic surgery [13, 14]. Medium- and long-term follow-up data were obtained through the same medical database. The patients excluded from the study were those who were not suitable for major operations, or affected by disseminated disease.

A complete preoperative diagnostic assessment, that in some cases included endoscopic retrograde cholangiopancreatography (ERCP) with brushing for cytology or fine needle transduodenal ultrasonography (US) guided endoscopic biopsy, was carried out for all patients. The administration of antibiotic and antithrombotic prophylaxis was routinely done and the same experienced surgeons performed the surgical procedure in both center. The confirm of tumor resectability, as well as the definition of the texture of pancreatic parenchyma (soft/fragile or hard/fibrotic), were intraoperatively obtained. On definitive pathological evaluation the absence of fibrosis or pancreatitis confirmed the definition of soft/fragile parenchyma.

Considering the age, patients were divided into two groups: 21 patients of less than 65 years (Group A) and 23 patients of more than 65 years (Group B). Comorbidities and complication rates were compared between the two groups. Indications to pancreatic duct closure were based on three main factors: main pancreatic duct size, texture of pancreatic remnant and personal experience of surgeon. Two tubular non-aspirative rubber drains were left inside (up to anterior pancreatic surface and posteriorly to biliary anastomosis) in all cases. In all 44 patients (group A and group B), the pancreatic stump was closed by a linear stapler (GIA60-80 Ethicon Inc, Somerville, New Jersey, USA, or Tyco^®^ GIA™ 60-80 or Tyco^®^ TA™60-90, Priceton, New Jersey, USA). Injection of octreotide (Longastatina 0.1 mg, Italfarmaco S.p.a., Milano, Italy) was initiated during operation and continued until postoperative day 7 (0.1 mg three times a day). Mortality and morbidity were considered within 30 days of the operation or during hospital stay. PF and postoperative bleeding were defined according to International Study Group of Pancreatic Fistula criteria by measurements of amylase concentration in the drain fluids, systematically performed during postoperative days [15]. In patients with a PF conservative strategy was preferred as first choice with total parenteral nutrition, antibiotics-targeted therapy, administration of somatostatin analogues and percutaneous drainage. Reoperation was reserved in case of visceral perforation, deteriorating general conditions, sepsis, intra-abdominal collections intractable by percutaneous drainage or bleeding after failure (or contraindication) of radiological endovascular procedures.

To evaluate the presence of fluid collections in the abdomen, US or CT scan examinations were postoperatively performed. Percutaneous drainages were recommended for 4–5 cm-larger collections. A drainage of bilious fluid >50 mL/24 h was defined as a biliary leak. In the presence of infection and systemic inflammatory response a diagnosis of sepsis was made. Blood transfusions were recommended when hemoglobin level was <8 g/dL. According to American Diabetes Association, postoperative diabetes was defined [16]. The American Joint Committee on Cancer (AJCC) TNM Classification of Pancreatic Cancer was utilized to define the tumor extent [17].

Standard PD including antrectomy was performed for all 44 patients (100%). A preoperative decompressive endoscopic stenting according to obstructive jaundice was positioned in 12 patients (27.2%). Postoperative collections with high value of amylase were drained in 8 patients with percutaneous drainage (18.1%). According to CONKO-001 study, adjuvant chemotherapy with gemcitabine (1000 mg/m^2^) was administrated to 21 patients (47.7%) [18].

### Statistic analysis

Data were analyzed using descriptive statistics: for the categorical variables, the Pearson Chi-squared (exact) test and, for the quantitative variables, the independent t Student test were used. Data were reported as the mean value ± standard error of the mean (SEM). All calculations were performed using the software package GraphPad Prism, Version 6.01 for Windows (GraphPad Software, San Diego, CA, USA). Our values were considered statistically significant if *p* was < 0.05.

## Results

20 men and 24 women were included in the study (male/female ratio = 0.83) with mean age of 59.4 ± 11 years. Comorbidities were similar in the two groups (Table 1). Preoperative bilirubin mean value was 5.83 ± 5 mg/dL.

**Table 1:** Comorbidities on 105 consecutive patients

Comorbidity	Group A 21 pts (<65 years)	Group B 23 pts (>65 years)
Obesity	3 (14.2%)	4 (17.3%)
Hypertension	10 (47.6%)	18 (78.2%)
COPD	4 (19.04%)	11 (47.8%)
Atrial fibrillation	3 (14.2%)	7 (30.4%)
Hypertensive heart disease	2 (9.5%)	2 (8.6%)
Ischemic heart disease	3 (14.2%)	6 (26.08%)
Diabetes mellitus	2 (9.5%)	5 (21.7%)
HCV+	3 (14.2%)	2 (8.6%)
Hypercholesterolemia	1 (%)	3 (13.04%)
Liver Cirrhosis	1 (4.7%)	1 (4.3%)
Jaundice mean value	4.67 mg/dL	8.34 mg/dL

In all 44 patients, the texture of the pancreas was found to be soft with a not-dilated duct (diameter <3 mm). Mean operative time (315 ± 91 min) and mean intraoperative blood loss (503 ± 213 mL) were similar in the two groups.

Definitive pathologic examinations confirmed all the diseases (Table 2).

**Table 2:** Pathology

Pancreatic adenocarcinoma	35
Tumor of papillae	3
Neuroendocrine carcinoma	2
Common bile duct cancer	2
Advanced gallbladder cancer	1
Intraductal papillary mucinous neoplasms	1

The mortality rate was 2.2%; one patient of group A died from septic complications after perforation of transverse colon. Overall morbidity, considering also subclinical complications (postoperative PF grade A), was 73.6% for the entire series, respectively, 80.9% in group A and 74.2% in group B, with a difference not statistically significant.

A grade-A pancreatic fistula, which did not required further treatments, developed in 20 cases (45.4%; 13 in group A and 7 in group B; *p* < 0.05), grade-B in 5 patients (11.4%; 3 in group A and 2 in group B; statistically not significant) and a grade-C pancreatic fistula was observed only in one patient (2.2%; 1 in group A and 0 in group B).

Major reported postoperative complications were: delayed gastric emptying in 3 cases (6.8%; 2 in group A and 1 in group B, statistically not significant); abdominal abscess in 2 cases (4.5%; 1 in group A, 1 in group B, statistically not significant) mainly as a consequence of pancreatic fistula; biliary leakage in 3 patients (6.8%; 1 in group A, 2 in group B, statistically not significant) which were conservatively managed; acute pancreatitis in 3 cases (6.8%; 2 in group A and 1 in group B; statistically not significant); cholangitis in 2 cases (4.5%, 1 in group A and 1 in group B, statistically not significant); hemorrhage in 2 cases (4.5%; 1 in group A, 1 in group B, statistically not significant) (Table 3).

**Table 3:** Complications in separate groups (%)

Complication	Group A (n. 21)	Group B (n. 23)	*p* value	Odds Ratio
Pancreatic fistula	17 (13a/3b/1c)	9 (7a/2b/0c)	<0.05*	6.61
Delayed gastric emptying	2	1	0.7598	1.47
Abdominal abscess	1	1	0.9475	1.10
Hemorrhage	1	1	0.9475	1.10
Wound infection	5	6	0.8617	0.89
Biliary leakage	1	2	0.6051	0.53
Cardiovascular complication	2	7	0.0859	0.24
Respiratory complication	3	9	0.06456	0.26
Sepsis	1	1	0.2898	nd
Cholangitis	1	1	0.9475	1.10
Lymphatic fistula	0	0	n.d.	nd
Intestinal occlusion	0	0	n.d.	n.d.
Acute pancreatitis	2	1	0.7598	1.47
Pulmonary embolism	0	3	0.0864	0.0

The difference between mean hospital in group A (15 ± 7) and group B (23 ± 7) was statistically significant (*p* < 0.05).

## Discussion

As with any retrospective and not randomized review, this study has several limitations. The small number of patients and the different number of cases in the two groups (group B > group A) were the main bias. Anyway, the analysis of our results allows to make the following conclusions.

Twenty-six of 44 patients had a PF (59.1%) with soft pancreas and not-dilated pancreatic main duct (diameter <3 mm). Comparing the two groups, we observed a PF incidence of 80.9% (Group A) vs 39.1% (Group B). Therefore, in case of patients of more than 65 years undergone pancreatic stump mechanical closure the “elder” texture of pancreas seems to be a protective factor for PF.

Nevertheless, pancreatic duct closure was related with a high rate of grade-A fistulas, even if they were usually associated with negligible clinical sequelae. The grade-A fistulas were treated in most cases conservatively indeed, by percutaneous drains removed 1–6 months later. However, in some cases, prolonged hospitalization associated to higher costs was determined by PF and one group A patient died for septic complications. Definitely, an overall morbidity rate of 73.6% was reported, respectively, 80.9% in group A and 74.2% in group B (difference not statistically significant).

Even if derivative procedures must be preferred, pancreatic remnant stump closure may be considered in selected “high-risk” cases (soft/friable parenchyma with small pancreatic duct). Moreover, pancreatic stump closure is an easy alternative approach during Whipple’s procedure representing a useful “not-anastomotic” technique especially in elderly patients (>65 years) affected by comorbidities. In addition, it is a saving time procedure and does not influence the oncologic outcome and the overall survival.

When possible surgery represents the only potential cure for pancreatic cancer. PD is the pancreatic surgical procedure most frequently used for the treatment either of malignant or benign periampullary lesions as well. Unfortunately, at the time of diagnosis, only 20% of patients can be candidate to a radical surgical procedure. Perioperative mortality rate has decreased significantly in the last years, especially thanks to the improvements in the management of pancreatic remnant. However, morbidity still remains high, especially in centers with a low patient volume for pancreatic cancer [1]. The negative prognostic factors associated with high probability of cancer-related death within 1 year following radical surgery are: duration of symptoms >40 days, carbohydrate antigen 19.9 levels >200 U/mL and a poorly differentiated tumors (G3-4) [14]. Moreover, the major prognostic factors for relapsing disease are: finally cancer size, grading and nodal involvement. Therefore, neoadjuvant therapy should be considered in most cases in order to improve medium- and long-term results.

One of the most severe complication of pancreatic surgery is the anastomotic leakage following pancreatic anastomoses, (5–25% among series) and is often associated to life-threatening consequences (abdominal collection, sepsis and critical clinical conditions). Furthermore, the mortality rate is >10% when the pancreatic anastomosis is failed [7]. Moreover, PF is related to readmission, interventional radiology procedures, relaparotomy and delaying adjuvant chemio-radiotherapy with increases hospital stay, costs and mortality. In addition, the management of grade-C PF after failure of the conservative treatment still remains controversial [3, 7, 19]. In fact, completion pancreatectomy is unfortunately followed by substantial unfavorable metabolic side effects (“brittle” diabetes, malabsorption, osteopenia and diarrhea) [20, 21]. Consequently, several anastomotic techniques with their modifications have been described in order to prevent pancreatic leakage, with no uniform results [7, 22–26]. The main PF variables are: pancreatic texture, duct size, patient factors (especially age and comorbidities) and surgical factors (especially surgeon experience and skill, use of octreotide, pancreatic duct stenting, early drainage ablation, intra and postoperative bleeding). Definitely, duct size <3 mm, soft/fragile pancreatic parenchyma, low case volume of pancreatic resections, patients age and comorbidities significantly increase the risk of anastomotic leakage [7, 15, 25, 27–35].

The high incidence and potential severity of PF in PD have led some authors to re-propose the avoidance of any pancreatic anastomosis by closing/occluding the main duct, because a fistula from the over-sewn pancreatic remnant (without enzymatic activation) is less dangerous than one related to anastomotic procedures [8, 10, 11, 36–39]. Nevertheless, this technique was rarely used because of the risk of postoperative pancreatitis, permanent exocrine and endocrine insufficiency, the fear of severe hemorrhagic complications. It was successively abandoned or reserved to selected cases [40, 41]. However, several studies that compared different pancreatic anastomotic techniques with PD without any pancreatic anastomosis, reported a significantly decreased morbidity and mortality with the latest technique, particularly for elder people [42–49].

## Conclusions

PF following mechanical duct occlusion was nearly always reported, even if mostly with subclinical but not negligible sequelae. Therefore, according to the literature data, derivative procedures might be associated to lower postoperative morbidity and should be preferred. Regardless of the adopted surgical technique, PF occurred in almost the totality of patients with soft pancreas texture that was considered the main risk factor especially in elderly patients affected by comorbidities. Therefore, in selected “high risk” elderly patients (>65 years) closure of the pancreatic stump, as saving time procedure, can be a useful tool in the surgical armamentarium in order to reduce morbidity related to derivative techniques. In addition, it does not affect the oncologic rules and overall survival. In order to better evaluate PF risk factors in large homogeneous clinical series, prospective randomized controlled trials are highlighted.

## Abbreviations

PD: Pancreatoduodenectomy
PF: Pancreatic fistula
ERCP: Endoscopic retrograde cholangio-pancreatography
US: Ultrasonography
SEM: Standard error of the mean
